# Immune-Enhancement and Anti-Inflammatory Activities of Fatty Acids Extracted from *Halocynthia aurantium* Tunic in RAW264.7 Cells

**DOI:** 10.3390/md16090309

**Published:** 2018-09-01

**Authors:** Chaiwat Monmai, Seok Hyeon Go, II-Shik Shin, Sang Guan You, Hyungjae Lee, Seok Beom Kang, Woo Jung Park

**Affiliations:** 1 Department of Marine Food Science and Technology, Gangneung-Wonju National University, Gangneung, Gangwon 25457, Korea; bbuayy@gmail.com (C.M.); gogogo171717@gmail.com (S.H.G.); shinis@gwnu.ac.kr (I.-S.S.); umyousg@gwnu.ac.kr (S.G.Y.); 2 Department of Food Engineering, Dankook University, Cheonan, Chungnam 31116, Korea; lee252@dankook.ac.kr; 3 Citrus Research Station, National Institute of Horticultural and Herbal Science, RDA, Seogwipo 63607, Korea; hortkang@korea.kr

**Keywords:** *Halocynthia aurantium*, tunic, fatty acids, immunomodulation, NF-κB pathway, MAPK

## Abstract

*Halocynthia aurantium*, an edible ascidian species, has not been studied scientifically, even though tunicates and ascidians are well-known to contain several unique and biologically active materials. The current study investigated the fatty acid profiles of the *H. aurantium* tunic and its immune-regulatory effects on RAW264.7 macrophage cells. Results of the fatty acid profile analysis showed a difference in ratios, depending on the fatty acids being analysed, including those of saturated fatty acids (SFA), monounsaturated fatty acids (MUFA), and polyunsaturated fatty acids (PUFA). In particular, omega-3 fatty acids, such as eicosatrienoic acid n-3 (ETA n-3), eicosapentaenoic acid (EPA), and docosahexaenoic acid (DHA), were much higher than omega-6 fatty acids. Moreover, the *H. aurantium* tunic fatty acids, significantly and dose-dependently, increased the NO and prostaglandin E2 (PGE_2_) production in RAW264.7 cells, for immune-enhancement without cytotoxicity. In addition, these fatty acids regulated the transcription of immune-associated genes, including *iNOS*, *IL-1β*, *IL-6*, *COX-2*, and *TNF-α*. These actions were activated and deactivated via Mitogen-activated protein kinase (MAPK)and NF-κB signaling, to regulate the immune responses. Conversely, the *H. aurantium* tunic fatty acids effectively suppressed the inflammatory cytokine expressions, including *iNOS*, *IL-1β*, *IL-6*, *COX-2*, and *TNF-α*, in LPS-stimulated RAW264.7 cells. Productions of COX-2 and PGE_2_, which are key biomarkers for inflammation, were also significantly reduced. These results elucidated the immune-enhancement and anti-inflammatory mechanisms of the *H. aurantium* tunic fatty acids in macrophage cells. Moreover, the *H. aurantium* tunic might be a potential fatty acid source for immune-modulation.

## 1. Introduction

Lipids, including fat-soluble vitamins and fatty acids, are critical nutrients for human health and the prevention of diseases associated with its compositional change [1]. Free fatty acids (FFAs) have been known to be related to immune-modulation [2], and various other fatty acids, such as saturated fatty acids (SFAs), monounsaturated fatty acids (MUFAs), and polyunsaturated fatty acids (PUFAs), are coupled with chronic diseases, including cardiovascular disease [3], cancers [4], and diabetes [5].

PUFAs, including omega-6 and omega-3 fatty acids, have been known to be associated with inflammation and the immune system [6]. Further, critical fatty acids among PUFAs, which contain more than 20 carbon atoms and more than two double bonds, are arachidonic acid (ARA, 20:4n-6), eicosapentaenoic acid (EPA, 20:5n-3), and docosahexaenoic acid (DHA, 22:6n-3). These play the roles of intermediates for lipid chemicals like eicosanoids and docosanoids, which are important cellular signaling molecules related to inflammation and immune regulation in physiological systems. They are produced by the catalysis of cyclooxygenases (COX) and lipoxygenases (LOX) [7,8,9]. The precursors of eicosanoids, ARA and EPA, are associated with pro-inflammation and anti-inflammation [10,11], and DHA, which is the precursor of anti-inflammatory docosanoids, such as resolvins and protectins, is a lipid intermediate for cancer therapy. Moreover, EPA, DHA, and long-chain omega-3 PUFA, have been reported to have beneficial effects in diseases, such as arthritis and asthma, through the use of positive immune-regulation [7,9].

Tunicates and ascidians are well-known to contain several unique and biologically active materials, including lipids and lipophilic compounds [12]. One of the sea squirts, *Halocynthia roretzi*, is known to hold various functional components, such as eicosapentaenoic acid (EPA), docosahexaenoic acid (DHA), carotenoids, taurine, and plasmalogen [13]. The *H. roretzi* tunics have also been reported to contain high amounts of carotenoid and other nutritious substances [14]. In a report by Konishi [15], acetylene carotenoids, such as alloxanthin and diatoxanthin, which are contained in lipids of *H. roretzi*, suppressed pro-inflammatory cytokine secretions (such as *IL-6* and *IL-1β*) from macrophage-like RAW264.7 cells, that have been stimulated by lipopolysaccharides. It was also reported that halocynthiaxanthin extracted from *H. roretzi* exhibited an anti-proliferative effect on various tumor cells [16].

In contrast, functional fatty acids extracted from *Halocynthia aurantium* which is an edible ascidian species, have not been studied as much as *Halocynthia roretzi*, even though *H. aurantium* is a valuable organism of the benthic marine population in the northern region of the East Sea, Korea [17]. In particular, *H. aurantium* fatty acids have not reported to be associated with immune-regulation, in any scientific study, to date. Therefore, the current study investigated the immunomodulation effects of tunic fatty acids extracted from *H. aurantium*, on the macrophage cell RAW264.7.

## 2. Results

### 2.1. Fatty Acid Analysis of Halocynthia aurantium Tunic

**Figure 1:**
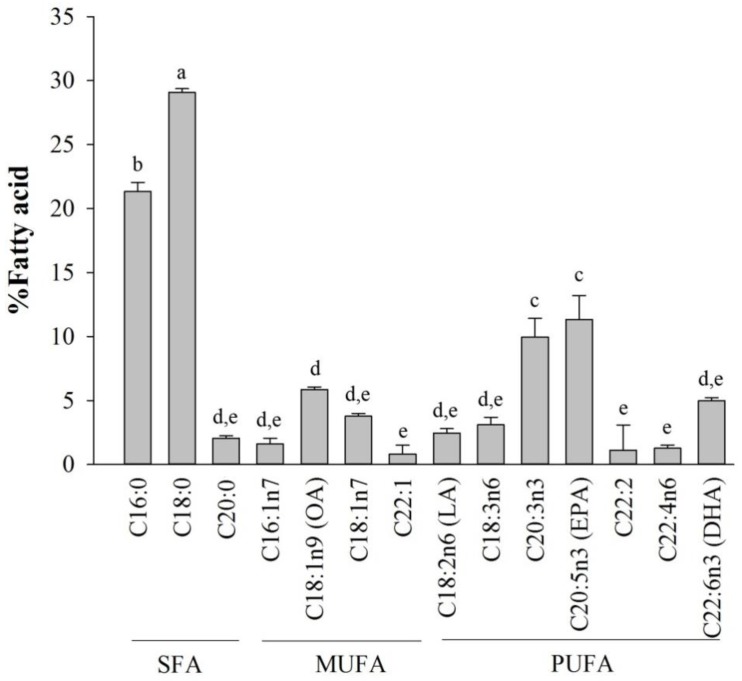
Fatty acid composition of tunic from *Halocynthia aurantium*. Data are presented as means ± standard deviation (*n* = 5). The letters a, b, c, d, e indicate a significant difference (*p* < 0.05) between the amount of fatty acid (where, a > b > c > d > e).

The total fatty acid compositions from the tunic of sea squirts are shown in Figure 1. The fatty acids were divided into three groups of saturated (SFAs), monounsaturated (MUFA), and polyunsaturated (PUFA) fatty acids. SFAs comprised 53.17 ± 1.36%, and unsaturated fatty acids, including MUFAs and PUFAs, comprised 12.18 ± 0.86% and 34.65 ± 1.81%, respectively. Among the SFAs, palmitic acid (16:0) and stearidonic acid (18:0) were highest, similar to that of mammals, such as, humans, mice, and other species. Additionally, oleic acid (OA, 18:1) was the highest MUFA, and the tunic contained a much higher proportion of omega-3 PUFAs, such eicosatrienoic acid (ETA, 20:3n-3), EPA and DHA, than omega-6 PUFAs, especially so, for EPA, which was ranked as the highest PUFA.

### 2.2. Effect of Fatty Acids from H. aurantium, Tunic Cytotoxicity and NO Production on Macrophages

**Figure 2:**
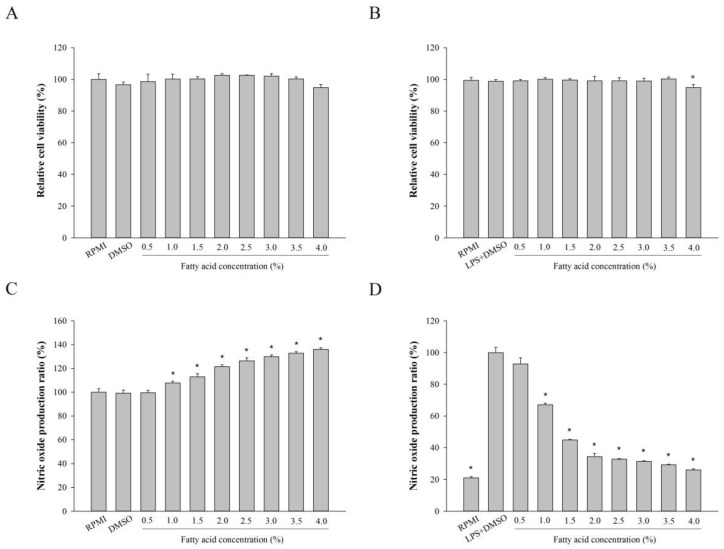
The cytotoxic effect and NO production of fatty acids from the *H. aurantium* tunic. (**A**) The cytotoxic effect of *H. aurantium* fatty acids on macrophage proliferation in RAW264.7 cells; (**B**) The cytotoxic effect of *H. aurantium* fatty acids on macrophage proliferation in LPS-stimulated RAW264.7 cells; (**C**) The effect of *H. aurantium* fatty acids on nitric oxide production in RAW264.7 cells; (**D**) The effect of *H. aurantium* fatty acids on nitric oxide production in LPS-stimulated RAW264.7 cells. RAW264.7 cells were stimulated with or without 1 μg/mL of LPS for 24 h. Significant differences are *p* < 0.01, compared with dimethyl sulfoxide (DMSO) or LPS (*).

To investigate the cytotoxicity of tunic fatty acids extracted from *H. aurantium*, RAW264.7 cells were cultured with different concentrations of fatty acids. Cellular viability, along with the fatty acids, are shown in Figure 2A, in which tunic fatty acids did not provide any toxicity to RAW264.7 macrophage cells for up to 4%.

In addition, we analyzed the potential immune-stimulation properties and anti-inflammatory effects of fatty acids on RAW264.7 cells when the cells were producing nitric oxide (NO).This is an important factor for pathophysiological conditions, such as inflammation and infection [18]. First, Figure 2B shows that tunic fatty acids enhanced NO production depending on the fatty acid concentrations. Figure 2C displays the anti-inflammatory activity of fatty acids when lipopolysaccharide (LPS) was used to stimulate inflammation. The results showed that tunic fatty acids significantly inhibited LPS-stimulated NO production depending on the fatty acid concentrations, and NO productions were lowest, and similar, between 2.0–4.0% fatty acid concentrations.

### 2.3. Effect of Fatty Acids from H. aurantium Tunic on Immune Gene Expressions

**Figure 3:**
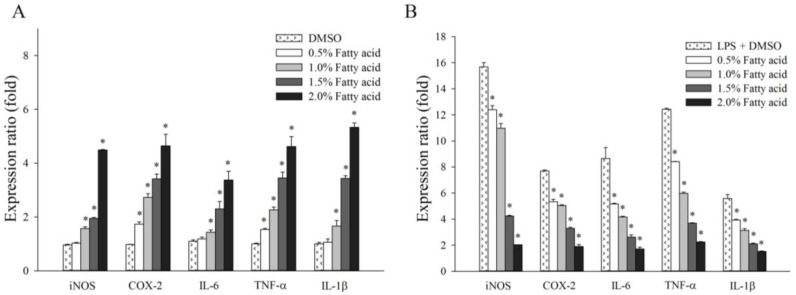
Quantification of immune genes in relative expression (fold). (**A**) Relative expression of *H. aurantium* fatty acids in RAW264.7 cells; (**B**) Relative expression of *H. aurantium* fatty acids in LPS-stimulated RAW264.7 cells. Significant differences are *p* < 0.01 compared with DMSO or LPS (*).

Figure 3 shows the immune-regulation of the *H. aurantium* tunic fatty acids via immune-associated gene expression in RAW264.7 cells. First, gene expressions in RAW264.7 cells increased in a dose-dependent manner, depending on the fatty acid concentration, without any LPS-treatment. Most gene expressions were similarly stimulated by the fatty acids (Figure 3A). However, in LPS-stimulated RAW264.7 cells, the expression of immune-associated genes was dose-dependently inhibited, depending on the fatty acid concentrations (Figure 3B). In particular, the expression of *iNOS*, one of the key enzymes that can catalyze NO production, was highly reduced by tunic fatty acids, and the expression of other cytokine genes, such as *IL-1β*, *IL-6* and *COX-2*, were also dose-dependently decreased. In addition, the expression of *TNF-α*, another well-known inflammatory biomarker for immune-regulation [19,20], was highly and dose-dependently down-regulated.

### 2.4. Effect of Fatty Acids from H. aurantium Tunic on MAPK and NF-κB Signaling Pathways

**Figure 4:**
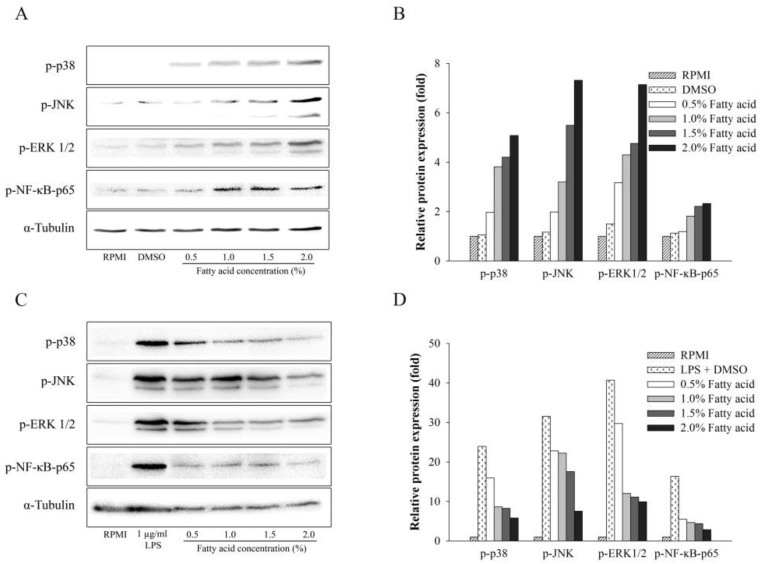
The effect of *H. aurantium* fatty acids on proteins associated with NF-κB and MAPK pathways. (**A**) Western blot of proteins from RAW264.7 cells; (**B**) Relative band intensity of proteins from RAW264.7 cells; (**C**) Western blots of proteins from LPS-stimulated RAW264.7 cells; (**D**) Relative band intensity of proteins from LPS-stimulated RAW264.7 cells.

In order to determine the signaling routes by which the *H. aurantium* tunic fatty acids modulate immune-enhancement and anti-inflammation, immune signaling pathways, such as NF-κB and MAPK, were investigated. As shown in Figure 4A,B, *H. aurantium* tunic fatty acids dose-dependently activated the phosphorylation of NF-κB p-65 of the NF-κB signaling pathway, indicating that phosphorylated NF-κB protein translocated into the nucleus to activate this pathway. Similar to NF-κB signaling activation, *H. aurantium* tunic fatty acids enhanced the phosphorylation level of MAPK proteins, such Extracellular signal–regulated kinases 1/2 (ERK1/2), c-Jun N-terminal kinase JNK, and p38, in a dose-dependent manner, indicating that MAPK signaling was activated. In contrast, *H. aurantium* tunic fatty acids strongly inhibited NF-κB p-65 phosphorylation of the NF-κB signaling pathway under LPS-stimulation in macrophage cells. Moreover, those fatty acids inhibited ERK1/2, JNK, and p38 phosphorylation in a dose-dependent manner in LPS-stimulated RAW264.7 cells (Figure 4C,D). These results indicated that *H. aurantium* tunic fatty acids suppressed inflammation in LPS-stimulated macrophage cells through NF-κB and MAPK signaling routes.

### 2.5. Effect of Fatty Acids from H. aurantium Tunic on PGE_2_ Levels

**Figure 5:**
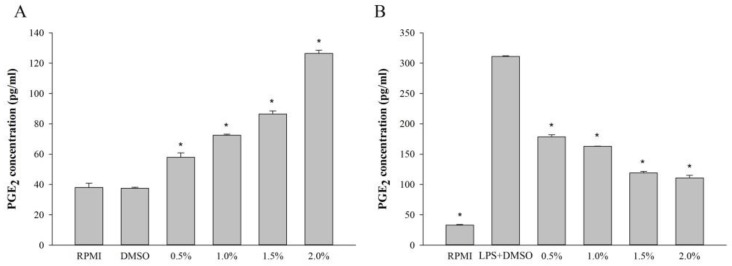
Quantification of PGE_2_ production from (**A**) RAW264.7 cells and (**B**) LPS-stimulated RAW264.7 cells. Significant differences are *p* < 0.01 compared with DMSO or LPS (*).

The immunomodulatory and anti-inflammatory effects of *H. aurantium* tunic fatty acids on RAW264.7 cells, were evaluated by the production of PGE_2_, one of the critical immune biomarkers [21]. Figure 5A shows that the production of PGE_2_ was dose-dependently increased according to the concentration of *H. aurantium* tunic fatty acids. On the contrary, PGE_2_ production was dose-dependently inhibited, depending on the concentration of *H. aurantium* tunic fatty acids, in LPS-stimulated RAW264.7 cells (Figure 5B).

## 3. Discussion

Various sea squirts are known to be valuable organisms of the benthic marine population which contains many functional components, including eicosapentaenoic acid (EPA), docosahexaenoic acid (DHA), carotenoids, taurine, and plasmalogen [13]. This current study aimed to investigate the immunomodulatory and anti-inflammatory activities of fatty acids extracted from one of edible sea squirt, *H. aurantium*, on a type of murine macrophage, RAW264.7 cells. The results showed the fatty acid profiles of *H. aurantium* tunic and their effects on immune systems in RAW264.7 cells for immune-stimulation and anti-inflammation.

Among tunic fatty acids, the most abundant fatty acids were SFAs, such as stearic (18:0) and palmitic (16:0), which are similar to the report by Fomenko et al. [22]. It was reported that these SFAs enhanced pro-inflammatory cytokines and immune signaling pathways [23], indicating that SFAs from *H. aurantium* tunic might play a role in immune-enhancing effects in RAW264.7 cells. Further, PUFAs were much higher than MUFAs, including palmitoleic acid (16:1) and OA, in the *H. aurantium* tunic and omega-3 fatty acids, such as ETA, EPA, and DHA, were much higher than omega-6 fatty acids. Long chain omega-3 fatty acids have beneficial effects on systemic inflammation via disruption of the toll-like receptor (TLR)-signaling cascade and the production of anti-inflammatory eicosanoids, which are mediated by their incorporation into the plasma membrane [24,25]. Furthermore, the action of EPA and DHA on inflammation has been known to affect the phospholipid fatty acid compositional change in the cell membrane, disturbance of lipid rafts, and inactivation of NF-κB, resulting in an alteration of the gene expression associated with immune and fatty acid metabolism [11]. This indicates that the tunic may be an important organ for the use of EPA and DHA, which are precursors for anti-inflammatory eicosanoids and docosanoids and can regulate anti-inflammatory activation [26].

Macrophages are mainly associated with acute and chronic inflammatory responses, which stimulate NO generation, to enhance macrophage functions of killing microorganisms [27]. Therefore, the fatty acids extracted from *H. aurantium* tunic were investigated for immune-stimulation and anti-inflammation using RAW264.7 cells. Figure 2A shows no cytotoxicity of those fatty acids on RAW264.7 cells, suggesting that these are safe at a concentration of up to 4.0% of fatty acids. In addition, the productions of NO and PGE_2_, which are critical immune-regulatory biomarkers for pain, fever, swelling, and tenderness [28], were examined in RAW264.7 cells. The results displayed that NO and PGE_2_ were significantly increased according to the fatty acid concentrations (Figure 2B and Figure 4A). However, NO and PGE_2_ were significantly and dose-dependently decreased in LPS-stimulated RAW264.7 cells (Figure 2C and Figure 4B). This suggests that the *H. aurantium* tunic fatty acids not only enhanced the levels of immune biomarkers for immune-enhancement, but they also decreased NO production for anti-inflammation in macrophage cells.

In addition to the production of NO and PGE_2_, the expression of immune-associated genes, such as *IL-1β*, *IL-6*, and *TNF-α* that are related to inflammation, have been known to regulate immune systems in macrophages [29]. To control immune systems, NF-κB cooperates with inflammatory mediators, such as *iNOS* and *COX-2*, and pro-inflammatory cytokines [30]. The activation of NF-κB is stimulated via the phosphorylation and degradation of IκBα in order to modulate inflammatory action [31]. Moreover, MAPK, including ERK1/2, JNK, and p38, is crucial for the regulation of cell growth and for cellular differentiation, and is controlled by cellular reactions of immune factors, such as cytokines and stresses [32]. Further, MAPK signaling has been thought to attenuate NF-κB activation, stimulating the expression of pro-inflammatory cytokines and inflammatory processes [33,34].

The current study showed the expression of immune associated genes, such as *iNOS*, *IL-1β*, *IL-6*, and *TNF-α* as well as *COX-2*, were significantly and dose-dependently increased in RAW non-LPS-treated 264.7 cells (Figure 4A) and decreased in LPS-stimulated RAW264.7 cells (Figure 4B). Varying the gene expressions triggered activation of NF-κB p-65 and MAPK, including ERK1/2, JNK, and p38. Consequently, the immune responses for both immune-enhancement (Figure 5A,B) and anti-inflammation (Figure 5C,D) were regulated, indicating that *H. aurantium* tunic fatty acids control immune regulation via MAPK as well as NF-κB signaling activation [35,36,37].

## 4. Materials and Methods

### 4.1. Halocynthia aurantium Sample

One of the sea squirts, *H. aurantium*, was obtained from the East Sea near Gangwon Province, South Korea. *H. aurantium* tunic was separated and collected.

### 4.2. Fatty Acid Extraction and Analysis

The extract of total fatty acid from the sea squirt tunic amounted to approximately 3.33% of the input raw material (4.5 g of raw material: 0.15 g of fatty acid yield). The extracted fatty acid was dissolved in DMSO to achieve a final concentration of 30 mg/mL for the immunomodulatory activity assays. Fatty acids were extracted and prepared according to the method of Garces and Mancha [38]. Fatty acid methyl ester (FAMEs) was made by the modified one-step hydrolysis, extraction, and methylation method previously described [39]. Prepared FAMEs from tunic of *H. aurantium* were analyzed by Gas chromatography (GC)—Flame ionization detection (FID) (Perkin Elmer, Waltham, MA, USA). Eicosanoic acid (20:0) was used as an internal standard in this study.

### 4.3. Macrophage Proliferation and Nitric Oxide Production

Murine macrophages, RAW264.7 cells, in Roswell Park Memorial Institute (RPMI)-1640 medium (supplemented with 10% Fetal Bovine Serum (FBS) and 1% penicillin/streptomycin) at a concentration of 1 × 10^5^ cells/mL were seeded in a 96-well plate. After 24 h, the different concentrations of tunic fatty acids (0.5%, 1.0%, 1.5%, 2.0%, 2.5%, 3.0%, 3.5%, or 4.0%) were injected and incubated for another 24 h. All experiments were carried out in triplicate. EZ-Cytox Cell Viability Assay Kit (Daeil Lab service, Chungcheongbuk, Korea) was used to analyze cellular proliferation, as described by Kim et al. [40]. The cellular proliferation ratio (%) was calculated based on the following formula:

$$\mathrm{macrophage}\;\mathrm{proliferation}\;\mathrm{ratio}\left(\%\right)=\frac{\mathrm{the}\;\mathrm{absorbance}\;\mathrm{of}\;\mathrm{the}\;\mathrm{test}\;\mathrm{group}}{\mathrm{the}\;\mathrm{absorbance}\;\mathrm{of}\;\mathrm{the}\;\mathrm{control}\;\mathrm{group}}\times 100$$

Nitric oxide (NO) production was determined to analyze the immunomodulatory activity of fatty acids. Cells were pre-treated, with various concentrations of tunic fatty acids, for 1 h. Anti-inflammation cells were stimulated with LPS (from *Escherichia coli* O111:B4, Sigma-Aldrich), and immune-enhancement cells were treated without LPS for 24 h. Griess reagent (Sigma-Aldrich, St. Louis, MO, USA) was used for the analysis of the nitric concentration [41].

### 4.4. RNA Isolation and cDNA Synthesis

Total RNA was extracted from cells using the Tri reagent^®^ (Molecular Research Center, Inc., Cincinnati, OH, USA), and the RNA concentration was analyzed using the nanophotometer (Implen, München, Germany). First stand cDNA was synthesized using the High capacity cDNA Reverse Transcription Kit (Applied Biosystems, Waltham, MA, USA), according to the manufacturer’s instructions.

### 4.5. Expression Analysis of Immune Gene by Real-Time PCR

Quantification of RAW264.7 immune gene expressions was performed using the QuantStudio™ 7 Flex Real-Time PCR System (Thermo Fisher Scientific, Waltham, MA, USA) with a 96-well format in a total reaction volume of 20 µL/well of SYBR^®^ Premix Ex Taq™ II (Takara Bio Inc., Shiga, Japan). The reaction mixture consisted of 0.4 µM of each specific primer pair (Table 1) and 0.1 ng of cDNA templates. The results were calculated using the 2^−ΔΔC^_T_ method [42] and compared with β-Actin as a control gene of immune gene expressions.

### 4.6. Western Blot Assay

RAW264.7 cells were harvested with RIPA buffer (Tech & Innovation, Hebei, China) by stimulation with or without 1 µg/mL of LPS. The protein concentration was measured using the Pierce™ BCA Protein Assay Kit (Thermo Scientific, Waltham, MA, USA). After separation by SDS-polyacrylamide gel electrophoresis (SDS-PAGE), the proteins were transferred to a polyvinylidene fluoride (PVDF) membrane, and the western blot assay was performed as described by Narayanan et al. [43]. Specific antibodies were used for p-NF-κB p65 (Cell Signaling Technology), p-p38 (Cell Signaling Technology), p-ERK1/2 (Cell Signaling Technology), p-JNK (Cell Signaling Technology), and α-Tubulin (Abcam). Signals were recognized using the Pierce^®^ ECL Plus Western Blotting Substrate (Thermo Scientific, Waltham, MA, USA) and the blot was quantitated using the ChemiDoc XRS+ imaging system (Bio-Rad) and ImageLab software (version 4.1, Bio-Rad, Hercules, CA, USA).

### 4.7. Quantification of PGE_2_

PGE_2_ ELISA kit (Enzo Life Sciences, Farmingdale, NY, USA) was used to analyze PGE_2_ according to the manufacturer′s instructions. The analysis was performed in duplicate, and the quantification of PGE_2_ in the samples was evaluated based on a standard curve.

### 4.8. Statistics

‘Statistix (version 8.1)’ Statistics Software, Statistix, Tallahassee, FL, USA) was used for the statistical analysis, and the values were evaluated by one-way analysis of variance, followed by post-hoc Duncan’s multiple range tests. The differences between the two groups were compared using t-tests (*p* < 0.05). The results of fatty acid analyses are shown as means ± standard deviation. Differences between each fatty acid were examined for statistical significance using one-way analysis of variance, followed by Tukey’s pair-wise comparisons at *p* < 0.05.

## 5. Conclusions

Our study demonstrated the immune-enhancement as well as the anti-inflammatory regulation of *H. aurantium* tunic fatty acids (consisting of SFA, MUFA, and PUFA) in RAW264.7 cells. This is evident from the diverse biological results that could be observed, such as, alterations of the key, immune biomarkers like NO and PGE_2_, immune-associated gene expression, and, the MAPK and NF-κB signaling for regulating immune responses. These results might be helpful to understand the regulatory roles of *H. aurantium* tunic fatty acids, consisting of SFA, MUFA, and PUFA, on immune cells. Moreover, the results suggest that *H. aurantium* tunic has a potential fatty acid source which could be used for immune-modulation.

## Figures and Tables

**Table 1:** The sequences of oligonucleotide primers used for macrophage test of immune genes.

Gene	Accession No.	Sequence	Product Size (bp)
iNOS	BC062378.1	F: TTCCAGAATCCCTGGACAAG R: TGGTCAAACTCTTGGGGTTC	180
IL-1β	NM_008361.4	F: GGGCCTCAAAGGAAAGAATC R: TACCAGTTGGGGAACTCTGC	183
IL-6	NM_031168.2	F: AGTTGCCTTCTTGGGACTGA R: CAGAATTGCCATTGCACAAC	191
COX-2	NM_011198.4	F: AGAAGGAAATGGCTGCAGAA R: GCTCGGCTTCCAGTATTGAG	194
TNF-α	D84199.2	F: ATGAGCACAGAAAGCATGATC R: TACAGGCTTGTCACTCGAATT	276
β-Actin	NM_007393.5	F: CCACAGCTGAGAGGAAATC R: AAGGAAGGCTGGAAAAGAGC	193
